# mRuby, a Bright Monomeric Red Fluorescent Protein for Labeling of Subcellular Structures

**DOI:** 10.1371/journal.pone.0004391

**Published:** 2009-02-05

**Authors:** Simone Kredel, Franz Oswald, Karin Nienhaus, Karen Deuschle, Carlheinz Röcker, Michael Wolff, Ralf Heilker, G. Ulrich Nienhaus, Jörg Wiedenmann

**Affiliations:** 1 Institute of General Zoology and Endocrinology, University of Ulm, Ulm, Germany; 2 Department of Internal Medicine I, University of Ulm, Ulm, Germany; 3 Institute of Biophysics, University of Ulm, Ulm, Germany; 4 Boehringer Ingelheim Pharma GmbH & Co. KG, Biberach, Germany; 5 Department of Physics, University of Illinois at Urbana-Champaign, Urbana, Illinois, United States of America; 6 National Oceanography Centre, University of Southampton, Southampton, United Kingdom; Dartmouth College, United States of America

## Abstract

A monomeric variant of the red fluorescent protein eqFP611, mRuby, is described. With excitation and emission maxima at 558 nm and 605 nm, respectively, and a large Stokes shift of 47 nm, mRuby appears particularly useful for imaging applications. The protein shows an exceptional resistance to denaturation at pH extremes. Moreover, mRuby is about ten-fold brighter compared to EGFP when being targeted to the endoplasmic reticulum. The engineering process of eqFP611 revealed that the C-terminal tail of the protein acts as a natural peroxisomal targeting signal (PTS). Using an mRuby variant carrying the eqFP611-PTS, we discovered that ordered inheritance of peroxisomes is widespread during mitosis of different mammalian cell types. The ordered partitioning is realized by the formation of peroxisome clusters around the poles of the mitotic spindle and ensures that equal numbers of the organelle are inherited by the daughter cells. The unique spectral properties make mRuby the marker of choice for a multitude of cell biological applications. Moreover, the use of mRuby has allowed novel insights in the biology of organelles responsible for severe human diseases.

## Introduction

Fluorescent proteins (FPs) are powerful, specific marker tools for cellular imaging, and their range of applications is continuously expanding [1]. Red fluorescent proteins (RFPs) are of particular interest as they extend the color palette for multi-channel and FRET imaging, and the reduced scattering of long-wavelength light makes them attractive as markers for deep tissue imaging. A performance gap has been noticed for natural RFPs after isolation from marine invertebrates [2]–[4], in comparison to variants of the classical green fluorescent protein (GFP) [1]. In particular, their tendency to form dimers or tetramers can be detrimental for fusion marker applications [4]–[6]. Therefore, an entire “fruit basket” of monomeric FPs in many different hues was engineered from the tetrameric RFP DsRed [1], [2], [7], [8]. Together with monomeric variants of GFP, eqFP578 and several other anthozoan proteins, the emission colors of these FPs cover a wide range from blue to far-red [1], [5], [9]–[13]. Trans-cis isomerization of the chromophore induced by protein engineering of the *Entacmaea quadricolor* proteins eqFP578 and eqFP611 yielded bright far-red fluorescent markers with emission maxima shifted up to 639 nm [14]–[16]. Isomerization of the eqFP611 chromophore was also observed under intense illumination [17], commending this protein as a lead structure for the development of photoswitchable RFPs for superresolution microscopy [1], [18]. Enhanced RFPs are very attractive for use in biosensors that report on intracellular conditions via fluorescence resonance energy transfer (FRET) between two or more chromophores and complement the established FRET couples featuring yellow or orange fluorescent acceptor proteins [5], [9], [10], [12], [19]–[21]. A large Stokes shift, i.e., a large gap between excitation and emission peaks, assists in channel separation and facilitates the use of FPs in FRET-based applications and other multi-color imaging purposes. With excitation/emission maxima at 559/611 nm, eqFP611 offers itself as an ideal lead structure for the development of a monomeric RFP with a large Stokes shift, which is presented in the following.

## Results and Discussion

### Evolution of a monomeric eqFP611

**Figure 1:**
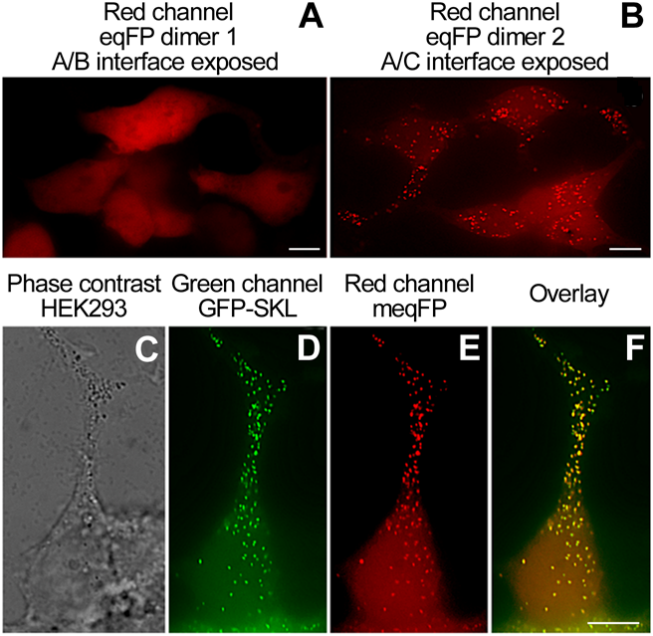
Subcellular localization of eqFP611 variants in HEK293 cells. (A) The eqFP dimer 1 (RFP611 T122R) [14] is evenly distributed over the cell. (B) The enhanced eqFP dimer 2 (T122G, F194A) shows a granular localization. (C–F) Cells co-expressing EGFP-SKL and an enhanced monomeric eqFP611 (T122R, F194A) variant show co-localization of green and red fluorescence in the peroxisomes. (C) Phase contrast image. (D) Green-channel image acquired with a standard FITC filter set. (E) Red-channel image produced with a standard TRITC filter set. (F) Overlay image of the green and red channels. Bars: 2.5 µm.

The F102I variant of eqFP611 was chosen as the starting material for the development of a monomeric variant owing to its excellent expression at 37°C [14]. Introduction of amino acids 122arginine and 194alanine, which were known to disrupt the A/B and A/C subunit interactions in tetrameric DsRed and eqFP611 [7], [22], resulted in a nearly complete loss of fluorescence. In several rounds of random and multi-site-directed mutagenesis, the fluorescence was recovered by replacing amino acids that apparently impeded proper folding and maturation of the chromophore. In HEK293 cells expressing these monomeric eqFP611 variants, the red fluorescence was not evenly distributed but rather appeared as dot-like structures in the cytoplasm. During mitosis, these spots were observed to congregate in two clusters and thereby were equally segregated between the daughter cells (supporting movies S1–S2 online). A similar effect was noticed for eqFP611 dimers that had the A/C interface disrupted, whereas dimers with disrupted A/B interface were evenly spread over the cell (Figure 1). This result suggested that exposure of the A/C interface may have made a subcellular targeting signal accessible. Indeed, sequence analysis revealed that amino acids 229GRL231 at the C terminus, and also the preceding triplet 226SKL228 may serve as a type-1 peroxisomal targeting signal (PTS) [23]–[26]. The presence of a functional PTS was verified by imaging HEK293 cells co-transfected with monomeric eqFP611 and EGFP-SKL, an established peroxisomal marker. As expected, perfect co-localization was obtained (Figure 1).

We succeeded in removing peroxisomal targeting by replacing the C-terminal sequence 222CDLPSKLGRL231 of eqFP611 by 222AGLGGG227. Amino acid modifications and even certain truncations did not suffice, suggesting that other residues also contribute to the signal sequence aside from the terminal triplet (Table S1 online). After completion of seven rounds of random mutagenesis and four rounds of multi site-directed mutagenesis, we finally obtained a bright red-fluorescent monomer, denoted as mRuby, which was characterized in detail. Compared to wild-type eqFP611, it contains altogether 28 amino acid replacements and is shorter by four amino acids (Figure S1 online). Finally, the codon usage of mRuby was optimized for mammalian expression systems. Analysed by flow cytometry, the fluorescence signal of living HEK293 cells expressing the codon optimized variant was 5–8 fold increased in comparison to the predecessor with unaltered codon usage but identical amino acid sequence (data not shown).

### Properties of mRuby

**Figure 2:**
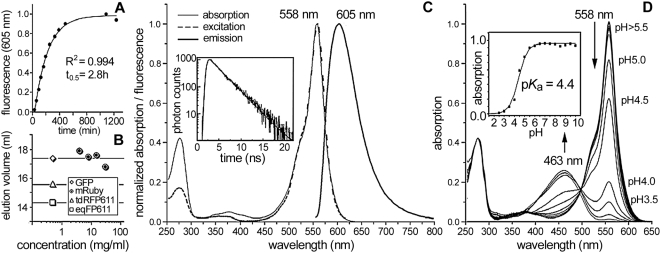
Characterization of mRuby properties. (A) *In vitro* chromophore maturation at 37°C as determined from the fluorescence emission at 605 nm (solid line: exponential fit). (B) Elution volume for concentrations of 4–30 mg/ml (0.16–1.2 mM) during gel-filtration analysis (standards for the oligomerization degree: GFP S65T, monomer; td-RFP611, dimer; eqFP611, tetramer). (C) Absorption, excitation and emission spectra (inset: fluorescence decay with a single exponential fit, yielding a lifetime of 2.6±0.1 ns). (D) Absorption spectra at various pH values (inset: absorption at 558 nm versus pH; Henderson-Hasselbalch fit reveals a pKa of 4.4).

At 37°C, fluorescence spectrometry on dilute solutions of the purified protein yields a half-maturation time of 2.8 h, which makes the protein suitable for most cell biological imaging applications (Figure 2A). A tendency to dimerize was absent in the entire range of physiologically relevant concentrations (Figure 2B). With excitation and emission maxima at 558 nm and 605 nm, respectively, mRuby combines red emission with a large Stokes shift (Figure 2C, Table 1). The fluorescence decay was fitted well by a single exponential, yielding a fluorescence lifetime of 2.6±0.1 ns. This fluorescence decay is comparatively long for a monomeric RFP [5], [27], and together with its high quantum yield (0.35) and molar extinction coefficient of 112,000 M^−1^ cm^−1^, mRuby appears as a superb marker in the red spectral range. It may be especially useful for FRET applications, where it bridges the gap between yellow-orange and far-red fluorescent proteins. The photobleaching probability of mRuby (5.3×10^−6^) is similar to the one of its predecessor RFP611 (4.9×10^−6^) [14] (Table 1). Surprisingly, the engineering process has significantly improved the stability of mRuby at pH extremes as compared to tetrameric RFP611 [14]. The mRuby chromophore protonates below pH 5, as indicated by the increasing absorption band at 463 nm (Figure 2D, Figure S2 online), but the protein remains stable even at pH 3. In contrast, the RFP611 chromophore decomposes quickly at pH 3, as witnessed by the appearance of a 390 nm absorption band indicative of the neutral GFP chromophore [14]. At pH 13, the fluorescence of a variety of monomeric FPs including EGFP disappears within seconds, whereas the fluorescence of mRuby takes about one hour to vanish completely (Figure S2 online).

**Table 1:** Fluorescence properties of EGFP compared to mRuby, its predecessors and other monomeric fluorescent proteins emitting in the orange to red spectral region.

FP Variant	Excitation Maximum [nm]	Emission Maximum [nm]	Stokes shift	QY	Emol [M^−1^ cm^−1^]	Relative Brightness^a^	Photobleaching Probability ×10^6^
EGFP	488	507	19	0.6	53,000^b^	1.0^b^	-
mKO [10]	548	559	11	0.6	51,600^b^	1.0^b^	-
mOrange [8]	548	562	14	0.69	71,000^c^	1.5^c^	-
TagRFP [5]	555	584	29	0.48	100,000^c^	1.5^c^	-
TagRFP-T [40]	555	584	29	0.41	81,000^c^	1.0^c^	-
mApple [40]	568	592	24	0.49	75,000^c^	1.2^c^	-
mRuby	558	605	47	0.35	not applicable^c^	1.2^d^	5.3
					112,000^d^		
eqFP611 [4]	559	611	52	0.45	116,000^c^	1.6^c^	6.5^e^
					146,000^d^	2.1^d^	
RFP611 [14]	559	611	52	0.48	120,000^c^	1.8^c^	4.9^e^
					151,000^d^	2.3^d^	
mCherry [8]	587	610	23	0.22	72,000^c^	0.5^c^	4.0^e^
					118,000^d^	0.8^d^	
mRaspberry [13]	598	625	27	0.15	86,000^c^	0.4^c^	-

a Product of QY and Emol compared to the brightness of EGFP (53,000 M^−1^ cm^−1^×0.6) [41].

b Concentration of the red chromophore deduced from the protein concentration as determined by colorimetric methods.

c Concentration of the red chromophore determined by the alkaline denaturation method [42].

d Concentration of the red chromophore determined by the *dynamic difference method* [14].

e Values from reference [14].

### Application tests

**Figure 3:**
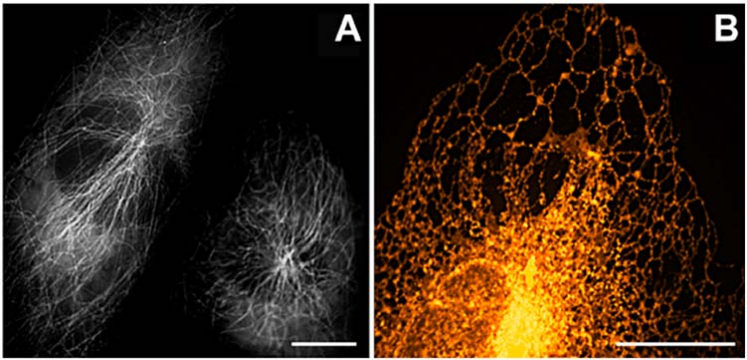
Applications of mRuby as a cellular marker protein. (A) Epifluorescence image of NIH3T3 cells expressing mRuby fused to human α-tubulin. Two cells from a single image were arranged in closer proximity (B) Spinning-disk confocal image of the endoplasmic reticulum of a HeLa cell stained with ER-mRuby-KDEL. False colors encode fluorescence intensity. Bars: 2.5 µm.

To examine the performance of mRuby as a marker in live-cell imaging applications, we fused it to the N-terminus of α-tubulin because imaging of microtubules with such a fusion construct is known to be very sensitive to oligomerization, aggregation tendency and C-terminal amino acid linker properties of the marker protein [8]. Figure 3A exemplifies an excellent performance of mRuby in highlighting tubulin fibers. Another challenging application, which requires a high stability of the marker protein, is its localization in the endoplasmic reticulum (ER). The construct ER-mRuby-KDEL allowed confocal imaging of the ER without difficulties (Figure 3B). Light exposure of only 0.4 s in the epifluorescence microscopy mode yielded excellent images, whereas the popular marker protein EGFP targeted to the ER (ER-EGFP-KDEL) required ∼ten-fold longer exposure times under comparable conditions (Figure S3 online).

### Imaging of peroxisomal inheritance

**Figure 4:**
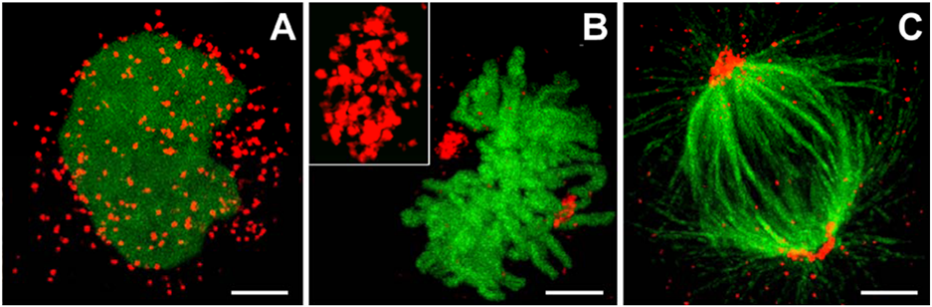
Peroxisome clusters in HEK293 cells. (A–C) Confocal images (Leica TCS 4Pi) of HEK293 cells co-expressing mRuby-PTS and H2B-EGFP during (A) interphase and (B) metaphase. The inset in (B) shows a magnification of a peroxisomal cluster. Tubulin fibers are labelled in green with Alexa Fluor©-488 via an anti-α-tubulin antibody (C) to reveal peroxisomal clustering at the spindle poles. Bars: 2 µm.

**Figure 5:**
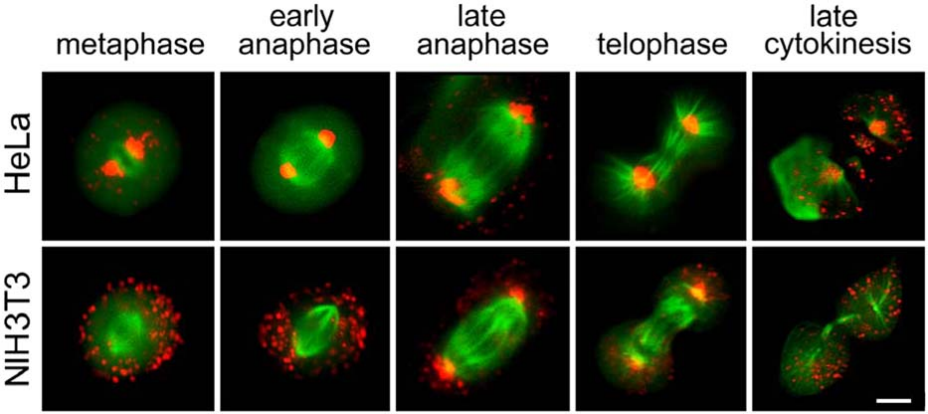
Inheritance of peroxisomes. Distribution of peroxisomes during different stages of mitosis in HeLa (upper row) and NIH3T3 cells (lower row). Peroxisomes are highlighted by mRuby-PTS. The spindle of the mitotic cells is stained with EGFP fused to a tubulin-binding protein [14]. Images were taken 24–48 h after transfection on an Olympus IX71 microscope equipped with standard FITC and TRITC filter sets. Bar: 5 µm.

Peroxisomes are the organelles that have been discovered last, and despite their essential functional roles in eukaryotic cells, such as fatty acid β-oxidation and hydrogen peroxide metabolism, many aspects of their biology are still not well understood [26]. Peroxisomes can be formed *de novo* from the endoplasmic reticulum [28], [29]. Controlled division and inheritance of peroxisomes during mitosis are further mechanisms helping to maintain stable cellular populations of this vital organelle [28], [30], [31]. Our observation with the early monomeric eqFP611 variant that highlighted two peroxisome clusters during mitosis of HEK293 cells suggests an ordered partitioning of this organelle to daughter cells. This finding contrasts the commonly held view that peroxisomes are distributed stochastically between dividing mammalian cells [32], [33]. Hence, we set out to further explore the issue of peroxisome inheritance in these cells. To this end, we restored the original C-terminal sequence of eqFP611 in mRuby to attain an efficient peroxisomal marker (mRuby-PTS). HEK293 cells were co-transfected with mRuby-PTS and the chromatin-associated histone 2B protein (H2B) fused to EGFP [1] so as to simultaneously image peroxisomes (in red) and chromatin (in green). During interphase, peroxisomes were seen to be evenly distributed over the cytoplasm (Figure 4A). However, at the beginning of metaphase, they began to congregate in two clusters in a distinct distance above and under the emerging metaphase plate (Figure 4B). Additional immunochemical staining of the mitotic spindle revealed that the peroxisomes clustered around the spindle poles (Figure 4C). We further examined this mechanism with two other mammalian cell lines, HeLa and NIH3T3. In HeLa cells, peroxisomes were also closely associated with the spindle poles during all stages of cell division (Figure 5). By contrast, NIH3T3 cells showed these peroxisomal associations to the centrosomes only during two stages of cell division, late anaphase and telophase. Interestingly, peroxisomes of plant cells display a completely different localization during the same stages of mitosis, forming ordered arrays in close association with the division plane [34]. Yet another distribution pattern was previously observed during division of yeast cells, where clustering of peroxisomes tends to be restricted mainly to the bud [35]. A potential mechanism for the formation of the mammalian peroxisome clusters might be that cells use microtubules of the mitotic spindle to direct the organelles towards the poles. Such a scenario appears possible as directional movement of peroxisomes guided by microtubules was previously described for mammalian cells [33]. By the ordered partitioning strategy, the cells can ensure that equal numbers of peroxisomes are inherited by the daughter cells. On the other hand, the distinct localization might indicate that peroxisomes fulfill a specific function during cell division [34]. Future work needs to reveal to which extend defects in the apparently widespread ordered inheritance of peroxisomes in mammalian cells are involved in the group of severe human diseases known as peroxisomal biogenesis disorders [31].

In summary, we have developed and characterized mRuby, a monomeric RFP with attractive properties for cellular imaging applications. By attaching the peroxisomal targeting sequence of eqFP611 to mRuby, we revealed that the peroxisomes of transfected mammalian cells were partitioned between the daughter cells in a highly ordered, cell line-specific manner, shedding new light on the biology of these organelles.

## Materials and Methods

### Mutagenesis and screening

The coding cDNA of eqFP611 and its variants were ligated in the pQE-32 vector (Qiagen, Hilden, Germany). Monomerizing interface mutations were introduced by site-directed mutagenesis via overlap extension PCR [36]. Random mutagenesis was performed according to the manufactureŕs protocol using the Diversify PCR Random Mutagenesis Kit (Clontech Laboratories, Inc., Mountain View, CA, USA), in conditions optimal for four to five mutations per 1000 bp. QuikChange Multi Site-Directed Mutagenesis Kit (Stratagene, La Jolla, CA, USA) was used subsequently to spot mutations that act synergistically to promote folding. For this purpose, up to six oligonucleotide primers were applied in the mutagenesis reaction to a mixture of up to four different mutant templates. *E. coli* XL1-Blue or XL10-Gold® ultracompetent cells (Stratagene, La Jolla, CA, USA) were transformed with the mutant plasmid libraries and bacterial colonies on agar plates were screened for fluorescence after overnight incubation at 37°C using a UVA (365 nm) transilluminator (Benda, Wiesloch, Germany) and a fluorescence microscope (Axioplan I, Carl Zeiss Jena GmbH, Jena, Germany).

### Protein expression, purification and gel filtration experiments

The eqFP611 variants carrying an N-terminal poly-His tag were expressed in *E. coli* M15 [pREP4] (Qiagen, Hilden, Germany). The proteins were purified using Talon™ metal affinity resin (Clontech Laboratories, Inc., Mountain View, CA, USA) and gel filtration (Superdex 200, Äkta System, GE Healthcare Biosciences, Little Chalfont, UK) as described [4], [22].

### Determination of maturation times

Maturation times at 37°C were determined as described [4], [14]. The protein solutions were diluted to an optical density of ∼0.1 at ∼560 nm after maturation to avoid artificially shortened maturation times due to inner filter effects. Maturation time courses at 37°C were recorded with a Cary Eclipse Spectrofluorometer (Varian Inc., Palo Alto, CA, USA).

### Spectroscopic characterization

Absorption spectra were recorded on a Cary 50 Spectrophotometer (Varian Inc., Palo Alto, CA, USA). Fluorescence emission and excitation spectra were measured with a Cary Eclipse Spectrofluorometer (Varian Inc., Palo Alto, CA, USA). The spectral characterization of mRuby at pH extremes and photobleaching experiments were conducted according to Ref. [14]. Fluorescence lifetime measurements on mRuby were performed with a Zeiss Axiovert 135 inverted microscope (Carl Zeiss Jena GmbH, Jena, Germany) by using a computer card for time-correlated single-photon counting (TIMEHARP 200, Pico-Quant, Berlin, Germany) as previously reported [4]. The excitation light pulses (532 nm) were generated by a mode-locked, frequency-doubled solid-state laser (GE-100, Time-Bandwidth Products, Zürich, Switzerland). The fluorescence quantum yield in 1× PBS, pH 7.0, was determined using eqFP611 (quantum yield of 0.45) as a reference [4].

Measurements of the molar extinction coefficient using the alkaline denaturation method [37] failed because of the extraordinary stability of mRuby. High concentrations of 0.3–1.0 N NaOH had to be applied to denature mRuby to determine the chromophore concentration by using the extinction coefficient of denatured GFP at pH 13 [37]. After complete denaturation, not only was the acylimine bond of the red chromophore reduced, but a significant fraction of the resulting GFP-chromophore was also destroyed, resulting in artificially high molar extinction coefficients of up to 250,000 M^–1^ cm^–1^. Consequently, the extinction coefficient of mRuby was measured with the *dynamic difference method* [14].

### Vector construction

For expression in mammalian cells, various eqFP611 variants were cloned into the pcDNA3 vector (Invitrogen, Carlsbad, CA, USA). Human α-tubulin from the pEGFP-Tub vector (Clontech Laboratories, Inc., Mountain View, CA, USA) was inserted into pcDNA3.1(-) downstream of the coding sequence of mRuby (Invitrogen, Carlsbad, CA, USA). An ER retention signal (KDEL) [38] was introduced to the C-terminus of mRuby and EGFP by PCR. The PCR products were inserted into the expression vector pCI-leader, encoding the signal peptide (METDTLLLWVLLLWVPGSTGD) from the murine Igκ chain.

### Eukaryotic expression and imaging

HeLa, HEK293 and NIH3T3 and cells were grown on chambered cover glasses (Nalge Nunc International Corp., Rochester, NY, USA). Images were taken 24–48 h post-transfection using fluorescence microscopes (DM IRB, Leica Microsystems, Wetzlar, Germany, IX71, Olympus, Hamburg, Germany) equipped with a digital camera (C4742, Hamamatsu, Hamamatsu, Japan), a 100-W mercury lamp and standard FITC (ex: HQ470/40; em: HQ525/50) and TRITC (ex: HQ545/30 em: HQ610/75) filter sets in combination with a dual band polychroic FITC/TRITC beam splitter (AHF, Tübingen, Germany). Subcellular localization of mRuby-PTS was assayed by confocal microscopy. HEK293 cells were transfected with a pcDNA3 vector encoding mRuby-PTS alone or in combination with a pcDNA3 vector encoding EGFP-H2B. Cells were fixed, permeabilized, and used directly for microscopic analysis or after immunostaining with an antiserum directed against human α-tubulin and an Alexa Fluor® 488-coupled secondary antibody.

Cells were plated on circular quartz coverslips (Leica Microsystems, Wetzlar, Germany). Sandwiches of two coverslips were assembled with PBS/glycerol (13%/87% by volume) as a mounting medium. Images were collected on a Leica TCS 4Pi scanning confocal laser microscope [39] (Leica Microsystems, Wetzlar, Germany) using a 100, NA 1.35, glycerol immersion objective and 488-nm excitation for EGFP and Alexa Fluor® 488, whereas mRuby was excited at 561 nm. The emitted photons were registered by using two avalanche photodiodes (APDs; SPCM-AQR-14, Perkin-Elmer, Canada). EGFP fluorescence was recorded in the green channel (500 nm–550 nm), whereas the emission of mRuby was detected in the red channel (607 nm–683 nm). A maximum-intensity projection of a 3D stack was prepared for presentation.

### Online Supporting Information

The Supporting Information consists of one supplementary table (table S1), three supplemental figures (figure S1, figure S2, figure S3). Two quick time movies (movie S1–movie S2) are provided showing the distribution of peroxisomes labelled with a monomeric eqFP611 variant in HEK293 cells during mitosis.

## Acknowledgments

We are grateful to R.Y. Tsien and co-workers (University of California, San Diego) for the donation of mFruits cDNA and to R. Schirmbeck (University of Ulm) for the pCI leader vector.

## Supporting Information

Figure S1 Supplemental Figure S1 online
(0.50 MB PDF)

Figure S2 Supplemental Figure S2 online
(0.15 MB PDF)

Figure S3 Supplemental Figure S3 online
(0.09 MB PDF)

Table S1 Supplemental table S1 online
(0.02 MB DOC)

Movie S1 Distribution of peroxisomes labelled with a monomeric eqFP611 variant in HEK293 cells during mitosis
(0.88 MB MOV)

Movie S2 Distribution of peroxisomes labelled with a monomeric eqFP611 variant in HEK293 cells during mitosis
(0.78 MB MOV)
