# Supplementary material for: mRuby, a Bright Monomeric Red Fluorescent Protein for Labeling of Subcellular Structures
Source: PLoS One. 2009 Feb 5;4(2):e4391. doi: 10.1371/journal.pone.0004391 (PMC2633614; doi:10.1371/journal.pone.0004391)
Supplement: Figure S2 — Supplemental Figure S2 online (0.15 MB PDF) [file pone.0004391.s002.pdf]

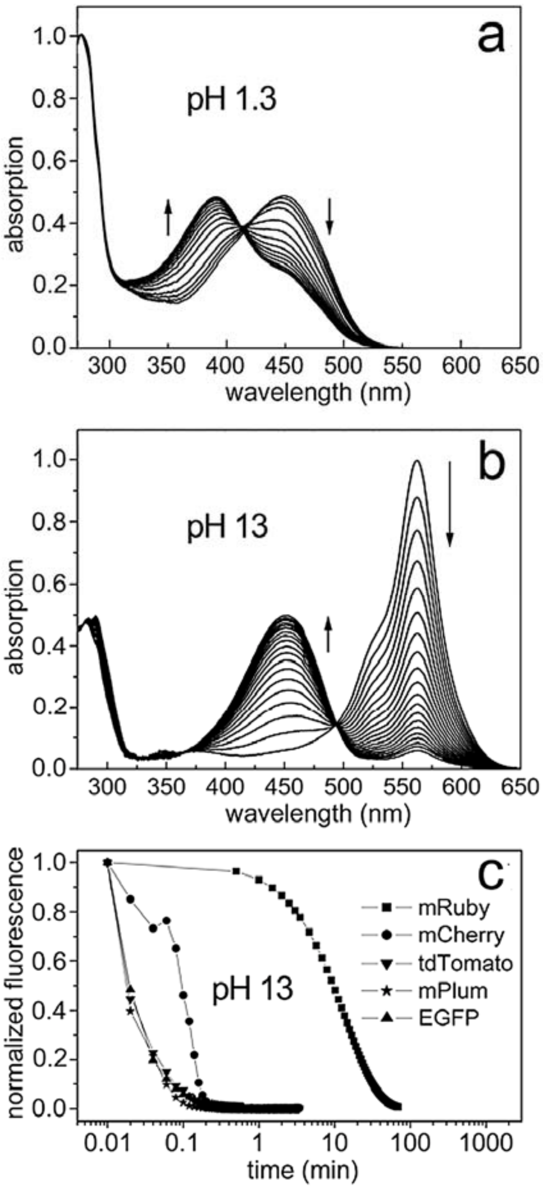

Supplemental Figure 2: Changes in the spectral properties of mRuby at pH extremes. (a) The increasing absorption at ~390 nm along with the decrease of the ~463 nm band of the protonated mRuby chromophore indicates the destruction of the acylimine bond of the chromophore over a period of 3.5 h time at pH 1.3. (b) Changes in the absorption spectrum of mRuby during a 1-h time course of alkaline denaturation at pH 13. (c) Time-dependent fluorescence decay of mRuby at pH 13 in comparison to other monomeric FPs, measured at their emission maxima.
