# Supplementary material for: mRuby, a Bright Monomeric Red Fluorescent Protein for Labeling of Subcellular Structures
Source: PLoS One. 2009 Feb 5;4(2):e4391. doi: 10.1371/journal.pone.0004391 (PMC2633614; doi:10.1371/journal.pone.0004391)
Supplement: Figure S3 — Supplemental Figure S3 online (0.09 MB PDF) [file pone.0004391.s003.pdf]

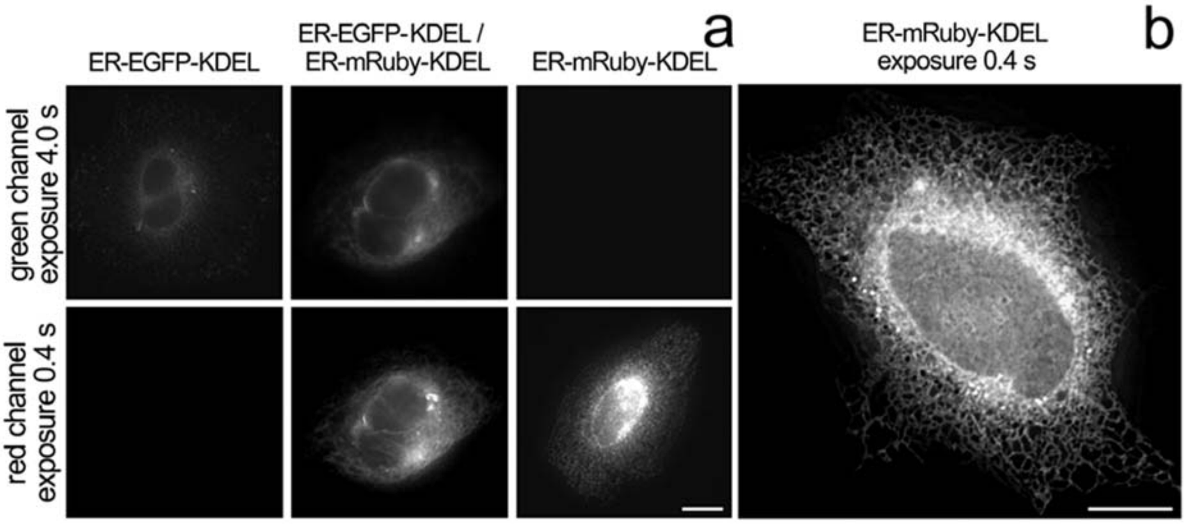

Supplemental Figure 3: Stability of mRuby targeted to the endoplasmic reticulum (ER). (a) Comparison of ER-staining in HeLa cells expressing ER-EGFP-KDEL (left column), ER-mRuby-KDEL (right column) or both (middle column). Imaging of the EGFP fluorescence in the green channel (upper row) required exposure times of 4.0 s, whereas mRuby fluorescence in the red channel could be already recorded with exposure times of 0.4 s using an Olympus IX71 microscope equipped with standard FITC (green channel) and TRITC (red channel) filter sets. (b) Magnified view of a HeLa cell expressing ER-mRuby-KDEL photographed on the Olympus IX71 microscope with TRITC filter. Bars: 2µm.
