# Supplementary material for: mRuby, a Bright Monomeric Red Fluorescent Protein for Labeling of Subcellular Structures
Source: PLoS One. 2009 Feb 5;4(2):e4391. doi: 10.1371/journal.pone.0004391 (PMC2633614; doi:10.1371/journal.pone.0004391)
Supplement: Table S1 — Supplemental table S1 online (0.02 MB DOC) [file pone.0004391.s004.doc]

Supplemental table 1: Peroxisomal localization of monomeric eqFP611 variants with different C-terminal sequences in HEK293 cells.

C-terminal sequence	Peroxisomal localization[a]	
	24 h posttransfection	48 h posttransfection
–$_{216}$HAVAKFCDLPSKLGRL$_{231}$[b]	++++++	++++++
–$_{216}$HAVAK**HSG**L**L**$_{225}$[c]	+++++ -	+++++ -
–$_{216}$HAVAK**HSG**L$_{224}$[c]	+++ - - -	+++ - - -
–$_{216}$HAVAKF**AG**L$_{224}$[c]	- - - - - -	++ - - - -
–$_{216}$HA**E**A**Q**F**SG**L**L**$_{225}$[c]	- - - - - -	+ - - - - -
–$_{216}$HAVAK**HSG**$_{223}$[c]	- - - - - -	- - - - - -
–$_{216}$HAVAK**HSGGG**$_{225}$[c]	- - - - - -	- - - - - -
–$_{216}$HA**E**A**Q**F**SG**$_{223}$[c]	- - - - - -	- - - - - -
$_{216}$HAVAKFCDL$_{224}$	- - - - - -	- - - - - -
–$_{216}$HAVAKF**AGGG**$_{225}$[c]	- - - - - -	- - - - - -
–$_{216}$HAVAKF**AG**L**GGG**$_{227}$[c,d]	- - - - - -	- - - - - -

[a] Rating based on the co-localization with EGFP-SKL from complete (++++++) to absent (- - - - - -)
[b] C-terminal sequence of wild-type eqFP611
[c] Residues deviating from the C-terminal sequence of wild-type eqFP611 are highlighted in red.
[d] C-terminal sequence of mRuby
