# Supplementary material for: mRuby, a Bright Monomeric Red Fluorescent Protein for Labeling of Subcellular Structures
Source: PLoS One. 2009 Feb 5;4(2):e4391. doi: 10.1371/journal.pone.0004391 (PMC2633614; doi:10.1371/journal.pone.0004391)
Supplement: Figure S1 — Supplemental Figure S1 online (0.50 MB PDF) [file pone.0004391.s001.pdf]

```
         ---------+---------+---------+---------+---------+---------+
eqFP611  MNS--------LIKENMRMMVVMEGSVNGYQFKCTGEGDGNPYMGTQTMRIKVVEGGPLP  52
mRuby    MNS--------LIKENMRMKVVLEGSVNGHQFKCTGEGEGNPYMGTQTMRIKVIEGGPLP  52
eqFP578  MSE--------LIKENMHMKLYMEGTVNNHHFKCTSEGERKPYEGTQTMKIKVVEGGPLP  52
TagRFP   MSE--------LIKENMHMKLYMEGTVNNHHFKCTSEGEGKPYEGTQTMRIKVVEGGPLP  52
mKate    MSE--------LIKENMHMKLYMEGTVNNHHFKCTSEGEGKPYEGTQTMRIKVVEGGPLP  52
DsRed    MRSSKN-----VIKEFMRFKVRMEGTVNGHEFEIEGEGEGRPYEGHNTVKLKVTKGGPLP  55
mRFP1    MASSED-----VIKEFMRFKVRMEGSVNGHEFEIEGEGEGRPYEGTQTAKLKVTKGGPLP  55
mCherry  MVSKGEEDNMAIIKEFMRFKVHMEGSVNGHEFEIEGEGEGRPYEGTQTAKLKVTKGGPLP  60

         ---------+---------+---------+---------+---------+---------+
eqFP611  FAFDILATSFMYGSKTFIKHTKGIPDFFKQSFPEGFTWERVTRYEDGGVFTVMQDTSLED  112
mRuby    FAFDILATSFMYGSRTFIKYPKGIPDFFKQSFPEGFTWERVTRYEDGGVITVMQDTSLED  112
eqFP578  FAFDILATSFMYGSKTFINHTQGIPDLFKQSFPEGFTWERITTYEDGGVLTATQDTSLQN  112
TagRFP   FAFDILATSFMYGSRTFINHTQGIPDFFKQSFPEGFTWERVTTYEDGGVLTATQDTSLQD  112
mKate    FAFDILATSFMYGSKTFINHTQGIPDFFKQSFPEGFTWERVTTYEDGGVLTATQDTSLQD  112
DsRed    FAWDILSPQFQYGSKVYVKHPADIPDYKKLSFPEGFKWERVMNFEDGGVVTVTQDSSLQD  115
mRFP1    FAWDILSPQFQYGSKAYVKHPADIPDYLKLSFPEGFKWERVMNFEDGGVVTVTQDSSLQD  115
mCherry  FAWDILSPQFMYGSKAYVKHPADIPDYLKLSFPEGFKWERVMNFEDGGVVTVTQDSSLQD  120

         ---------+---------+---------+---------+---------+---------+
eqFP611  GCLVYHAKVTGVNFPSNGAVMQKKTKGWEPNTEMLYPADGGLRGYSQMALNVDGGGYLSC  172
mRuby    GCLVYHAQVRGVNFPSNGAVMQKKTKGWEPNTEMMYPADGGLRGYTHMALKVDGGGHLSC  172
eqFP578  GCIIYNVKINGVNFPSNGSVMQKKTLGWEANTEMLYPADGGLRGHSQMALKLVGGGYLHC  172
TagRFP   GCLIYNVKIRGVNFPSNGPVMQKKTLGWEANTEMLYPADGGLEGRSDMALKLVGGGHLIC  172
mKate    GCLIYNVKIRGVNFPSNGPVMQKKTLGWEASTEMLYPADGGLEGRSDMALKLVGGGHLIC  172
DsRed    GCFIYKVKFIGVNFPSDGPVMQKKTMGWEASTERLYPRDGVLKGEIHKALKLKDGGHYLV  175
mRFP1    GEFIYKVKLRGTNFPSDGPVMQKKTMGWEASTERMYPEDGALKGEIKMRLKLKDGGHYDA  175
mCherry  GEFIYKVKLRGTNFPSDGPVMQKKTMGWEASSERMYPEDGALKGEIKQRLKLKDGGHYDA  180

         ---------+---------+---------+---------+---------+---------+
eqFP611  SFETTYRSKKTVENFKMPGFHFVDHRLERLEESDKEMFVVQHEHAVAKFCDLPSKLGRL   231
mRuby    SFVTTYRSKKTVGNIKMPGIHAVDHRLERLEESDNEMFVVQREHAVAKFAGLGGG       227
eqFP578  SFKTTYRSKKPAKNLKMPGFHFVDHRLERIKEADKETYVEQHEMAVAKYCDLPSKLGHR   231
TagRFP   NFKTTYRSKKPAKNLKMPGVYYVDHRLERIKEADKETYVEQHEVAVARYCDLPSKLGHK   231
mKate    NLKTTYRSKKPAKNLKMPGVYYVDRRLERIKEADKETYVEQHEVAVARYCDLPSKLGHK   231
DsRed    EFKSIYMAKKPVQ---LPGYYYVDSKLDITSHNEDYTIVEQYERTEGRHHLFL         225
mRFP1    EVKTTYMAKKPVQ---LPGAYKTDIKLDITSHNEDYTIVEQYERAEGRHSTGA         225
mCherry  EVKTTYKAKKPVQ---LPGAYNVNIKLDITSHNEDYTIVEQYERAEGRHSTGGMDELYK   236
```

Supplemental Figure 1. Multiple-sequence alignment of monomeric RFPs and their tetrameric predecessors. Modified amino acid residues are highlighted in different colors; cyan: residues involved in interface interactions; green: residues in the interior of the β-can; magenta: residues interacting with the chromophore; yellow: external residues; gray: GFP-termini of mCherry. The chromophore is underlined; β-strands are indicated by arrows.
